# Smartphone Use for Cervical Cancer Screening in Low-Resource Countries: A Pilot Study Conducted in Madagascar

**DOI:** 10.1371/journal.pone.0134309

**Published:** 2015-07-29

**Authors:** Rosa Catarino, Pierre Vassilakos, Stefano Scaringella, Manuela Undurraga-Malinverno, Ulrike Meyer-Hamme, Dominique Ricard-Gauthier, Juan Carlos Matute, Patrick Petignat

**Affiliations:** 1 Department of Gynecology and Obstetrics, Geneva University Hospitals, Geneva, Switzerland; 2 Geneva Foundation for Medical Education and Research, Geneva, Switzerland; 3 Saint-Damien Health-Care Center, Ambanja, Madagascar; State University of Maringá/Universidade Estadual de Maringá, BRAZIL

## Abstract

**Background:**

Visual inspection of the cervix after application of 5% acetic acid (VIA) is a screening technique for cervical cancer used widely in low and middle-income countries (LMIC). To improve VIA screening performance, digital images after acid acetic application (D-VIA) are taken. The aim of this study was to evaluate the use of a smartphone for on- and off-site D-VIA diagnosis.

**Materials and Methods:**

Women aged 30–65 years, living in the city of Ambanja, Madagascar, were recruited through a cervical cancer screening campaign. Each performed a human papillomavirus (HPV) self-sample as a primary screen. Women testing positive for HPV were referred for VIA followed by D-VIA, cervical biopsy and endocervical curettage according to routine protocol. In addition, the same day, the D-VIA was emailed to a tertiary care center for immediate assessment. Results were scored as either D-VIA normal or D-VIA abnormal, requiring immediate therapy or referral to a tertiary center. Each of the three off-site physicians were blinded to the result reported by the one on-site physician and each gave their individual assessment followed by a consensus diagnosis. Statistical analyses were conducted using STATA software.

**Results:**

Of the 332 women recruited, 137 (41.2%) were HPV-positive and recalled for VIA triage; compliance with this invitation was 69.3% (n = 95). Cervical intraepithelial neoplasia was detected in 17.7% and 21.7% of digital images by on-site and off-site physicians, respectively. The on-site physician had a sensitivity of 66.7% (95%CI: 30.0–90.3) and a specificity of 85.7% (95%CI: 76.7–91.6); the off-site physician consensus sensitivity was 66.7% (95%CI: 30.0–90.3) with a specificity of 82.3% (95%CI: 72.4–89.1).

**Conclusion:**

This pilot study supports the use of telemedicine for off-site diagnosis of cervical intraepithelial neoplasia, with diagnostic performance similar to those achieved on-site. Further studies need to determine if smartphones can improve cervical cancer screening efficiency in LMIC.

## Introduction

In Madagascar there are 3,194 new cervical cancer cases diagnosed every year and, according to country statistics, 1,804 women died of cervical cancer (estimations for 2012), making cervical cancer a leading cause of cancer death in females [1]. Cervical cancer remains the first most common female cancer in women aged 15 to 44 years in Madagascar [1]. In Western countries, cervical cancer can be prevented through cytology-based screening, but for low and middle-income countries (LMIC), large-scale screening is yet to be implemented, essentially because of a lack of human and material resources. To overcome barriers associated with the implementation of screening programs in LMIC, the World Health Organization has recommended cervical visual inspection with acetic acid (VIA), which is low cost, easy to perform and offers the possibility of immediate treatment, if needed [2].

Although VIA is well suited to LMIC, it has important limitations–namely subjectivity and lack of quality control [3, 4]. The positive predictive value of a positive test is also low (10–30%) [5, 6]. Good quality healthcare workers’ training is important for the success of the method [3]. Therefore, developing strategies to facilitate training and supervision for novice healthcare professional in underserved areas is essential to reduce false-positive rates and increase detection of real positive cases.

Recently, digital imaging after acid acetic application (D-VIA) has become an increasingly important tool for quality control [7]. Digital images of the cervix with or without magnification can be used immediately for better visualization of the cervix, thus presumably improving the diagnostic accuracy at the time of examination. This approach also allows a second diagnostic evaluation by an expert, in order to test the novice performance, as it is already done in many other medical contexts. Furthermore, digital images can be used for quality control as well as, for continuous education in e-learning platforms to help students across the globe to practice interpretation of VIA/VILI.

Although digital images may offer support for physicians, their downside is the need for digital cameras, which are expensive and require substantial field training to operate. New smartphones are an obvious choice for the development of "next-generation" tools for digital image acquisition. Smartphones are “easyuse” devices, allow immediate image capture and can send the images to a remote expert in real time.

In view of the growing applicability of this technology, the aim of this study was to evaluate smartphone use for on- and off-site D-VIA diagnosis and measure its diagnostic accuracy against histopathology.

## Materials and Methods

### Setting and study population

This study was conducted by the University of Geneva in collaboration with Madagascar’s Health and Family Planning Ministry and the Saint-Damien Healthcare Centre, in Ambanja, Madagascar. It is part of a long-term research project on cervical cancer prevention in Madagascar that aims to develop a cervical cancer screening and treatment approach for the country. Between January and August 2014, women were invited to perform a human papillomavirus (HPV) self-sampling test (self-HPV) as primary screening. Participants were recruited by advertising flyers, which were widely distributed. Upon arrival to the Saint-Damien Healthcare Centre, the women were checked for eligibility (aged between 30 and 69 years and able to sign an informed consent form), then asked to complete a self-HPV test, after which they completed a questionnaire to provide sociodemographic data and clinical information. Exclusion criteria were: having a positive history of cervical cancer or a total hysterectomy, presenting with any condition that could interfere with visualization of the cervix and being over 20 weeks pregnant.

For self-HPV, women were instructed to wash their hands before the procedure. Each participant received a package containing a specimen collection kit. The swab used for self-collection was a simple dry cotton swab. Recommendations were to hold the swab by the end of the handle, to insert the swab into the vagina until they met resistance, by avoiding contact with the external genitalia. Then, they should gently turn the swab three times, remove it, and insert it in its protective sleeve.

Genotyping for HPV was performed using the Anyplex II HPV28 detection test (Seegene, Seoul, South Korea), which simultaneously detects high and low-risk HPVs.

Women who were HPV-positive were invited to the Saint-Damien Healthcare Centre for further investigation.

This study was approved by the Cantonal Human Research Ethics Commission of Geneva (CCER, CER: 14–071) and the Malgasch National Commission for the Ethics of Science and Technology. A written consent form was signed by all participants.

#### Study procedure

Each HPV-positive woman underwent an initial speculum examination and visual cervical inspection without acetic acid by a trained on-site expert. Then, a conventional VIA/visual inspection with Lugol’s Iodine (VILI) was performed. In addition, the cervix was photographed by smartphone. Photographs were taken both before and after VIA/VILI (both termed in the text as “D-VIA”) by a medical student. The same day, D-VIA was emailed blindly to the University Hospitals of Geneva for immediate expert assessment. All women signed the informed consent form, giving permission for cervical image acquisition, transfer and later use of the images for educational purposes.

Biopsies of the cervix (at 6 o’clock and the lesion, if any) and endocervical curettage were performed as routine. Biological samples were sent to a Swiss laboratory for routine processing and histopathological diagnosis. Women deemed to be VIA positive were treated, if eligible [2].

#### Cervical image capture

Photos were taken at a distance of about 15 cm from the cervix, with 2× optical zoom. Image capture was conducted by using a smartphone (Samsung Galaxy S5), which has a 16 megapixels camera, with an aperture size of F2.2, focal length of 31 mm and a pixel size of 1.12 μm. The flash mode (LED) was permanently activated. The picture was always taken at the same approximate distance from the cervix, by using a universal digital camera support and bracket, where the smartphone was easily adjusted. Care was taken to avoid the intrusion of hair or the vaginal wall that would mask visualization of the cervix. Smartphone camera face-recognition system focus automatically on the cervix and not on other details.

All photographs were taken by a medical student, with no previous experience for VIA/VILI, who was trained for cervical image capture and assisted the physician during the examination. The training was performed in the previous weeks before departure to Madagascar and consisted in five sessions in the operating room with a gynecologist, where pictures of the cervix were taken using the same device and technique. During this training, images were obtained from patients who were going to undertake a conization procedure and who signed an informed consent form for image acquisition. These same images were used for educational purposes.

Basic knowledge of how to use a smartphone was required for cervical image capture.

Costs associated with the cervical image capture were about $580 US Dollar (Samsung Galaxy S5: $540, smartphone support: $40)

#### Telemedicine and diagnostic reliability

Three off-site physicians, who each have more than 5 years’ experience in colposcopy, working at the University Hospitals of Geneva analyzed and scored the D-VIA images. Results were scored as either normal D-VIA or abnormal D-VIA requiring immediate therapy or referral to a tertiary center. Each off-site physician was blinded to the result of the on-site physician and each gave an individual assessment, followed by a consensus diagnosis. Only the common assessment was emailed to the on-site physician.

#### Endocervical sample and cervical biopsies

An endocervical brush was used for endocervical sample collection and cervical forceps were used for biopsies. Both sample types were fixed in liquid formalin according to standard procedures. Histological results, the gold standard for diagnosis, were classified as negative, cervical intraepithelial neoplasia grade 1, 2 or 3 (CIN1, CIN2 or CIN3), or invasive carcinoma.

### Statistical analysis

Sensitivities and specificities, using cervical histology as the definitive result, were calculated for each specialist who had graded the photographs, as well as for the on-site expert. Positive and negative predictive values were also determined. Percentage of agreement was calculated for each pair of on-site results and for distant D-VIA results using McNemar’s test. Agreement of results was also assessed using Cohen’s kappa coefficient. Quantitative variables are expressed as means and standard deviations, and qualitative variables are expressed as percentages, unless otherwise stated. Data were analyzed with a statistical analysis software package (StataCorp.2013., Stata Statistical Software: Release 13. College Station, TX, USA).

## Results

### Participants' characteristics

A total of 332 women were recruited and performed self-HPV; of these, 137 (41.2%) were HPV-positive. The high-risk HPV types seen in participants of this study were 16, 18, 26, 31, 33, 35, 39, 45, 51, 52, 53, 56, 58, 59, 66, 68, 69, 73 or 82. Women testing positive for HPV were recalled to the Saint-Damien Healthcare Centre for further examination, although only 95 (69.3%) of the women responded. Table 1 summarizes the characteristics of these 95 women.

**Table 1:** Sample sociodemographic characteristics and past obstetric and gynecological history (n = 95)*

Variable	N° (%)
Age (mean ± sd), y	44.7±9.4
Age groups, y	
30–39	31 (33.0)
40–49	31 (33.0)
≥50	32 (34.0)
Marital Status	
Without a partner	24 (25.8)
With a partner	69 (74.2)
Education	
Unschooled	12 (12.6)
Primary education	39 (41.1)
Secondary education	42 (44.2)
Tertiary education	2 (2.1)
Work	
Employee with responsibilities	8 (8.4)
Employee with no responsibilities	3 (3.2)
Independent	17 (17.9)
Housewife	16 (16.8)
Farmer	30 (31.6)
Other	21 (22.1)
Age at menarche (mean ± sd), y	14.5±1.6
Age of first sexual intercourse (mean ± sd), y	16.5±2.6
Number of sexual partners, median (IQR)	5 (3–8)
Number of pregnancies, (mean ± sd)	5.1±3.0
Number of child (mean ± sd)	3.7±2.3
Age at first birth, median (IQR), y	18 (16–20)
Menopause	
Yes	38 (41.8)
No	53 (58.2)
Contraception:	
Pill	14 (15.2)
Injectable	22 (23.9)
Intrauterine device	1 (1.1)
None	55 (59.8)

Abbreviations: IQR = interquartile range; N. = number; sd = standard deviation; y = years.

*Some information from the 95 participants is missing

### Cervical cancer knowledge and awareness

Of the 95 women HPV positive women being evaluated by D-VIA, 39 (41.9%) had never heard of cervical cancer, 93 (97.9%) had never done a pap test, and 92 (96.8%) had never done an HPV test. Seven (7.7%) participants had someone in their family who had been diagnosed with cervical cancer. Cervical cancer awareness was mostly achieved through health centers, radio broadcasts or by social interaction (Table 2).

**Table 2:** Knowledge, awareness and history of cervical cancer and screening (n = 95)

Variable	N° (%)
Before today, have you ever heard of cervical cancer?	
No	39 (41.9)
Yes. Where?	54 (58.1)
Doctor/ Hospital	14 (25.9)
Radio	12 (22.2)
Family and friends	14 (25.9)
Women's association	5 (9.3)
Other	9 (16.7)
Was someone in your family diagnosed with cervical cancer?	
No	84 (92.3)
Yes	7 (7.7)
Have you ever done a pap test in your life?	
No	93 (97.9)
Yes	2 (2.1)
Have you ever done an HPV test in your life?	
No	92 (96.8)
Yes	3 (3.2)

Abbreviations: IQR = interquartile range; N. = number; sd = standard deviation; y = years.

### Histopathological analysis and on-site VIA evaluation and treatment

A total of eight cervical neoplasia cases were detected by histology: two CIN1, two CIN2, two CIN3 and two invasive cancers (Table 3). The on-site physician detected four out of six CIN2+ lesions, and treatment (electrocoagulation or conization) was proposed to these women. A missed cancer by the on-site physician was detected by the D-VIA consensus among physicians in Geneva, and since there were no signs of advanced invasion, a hysterectomy was proposed and accepted by the patient. Overall, two CIN2+ were missed and 18 healthy women were unnecessarily treated on-site.

**Table 3:** Sample analysis results by pathological histology and their diagnosis and treatment on site.

Patient	Hist.	Diagnosis On Site	Treatment On Site	Diagnosis N°1***	Diagnosis N°2***	Diagnosis N°3***	Consensus Diagnosis
N°1	CIN1*	No	None	Yes	Yes	Yes	Yes
N°2	CIN3*	Yes	Electrocoag.	Yes	No	No	No
N°3	CIN2*	Yes	None**	Yes	Yes	No	Yes
N°4	CA INV*	Yes	None**	Yes	Yes	Yes	Yes
N°5	CIN3*	Yes	Conization	No	Yes	Yes	Yes
N°6	CA INV*	No	Hysterect.	Yes	Yes	Yes	Yes
N°7	CIN2*	No	None	No	No	No	No
N°8	CIN1*	No	None	No	Yes	No	No

*CIN1, cervical intraepithelial neoplasia grade 1; CIN2, cervical intraepithelial neoplasia grade 2; CIN3, cervical intraepithelial neoplasia grade 3; CA INV, invasive carcinoma: Electroag. = Electrocoagulation; Hist. = Histology; Hysterect. = Hysterectomy

** Patient didn't show up for follow-up or refused treatment

*** Diagnosis N°1, diagnosed on photo by specialist N°1; Diagnosis N°2, diagnosed on photo by specialist N°2; Diagnosis N°3, diagnosed on photo by specialist N°3.

### Agreement between off-site experts and on-site physician

Overall, 17 cases were classified as VIA pathological by the on-site physician (17.7%). The off-site experts, using the digital images, identified 20 pathological VIA cases (21.7%). The inter-observer agreement in the assessment of VIA photography was fair (kappa 0.43) among the experts in Geneva. The agreement between the on-site physician and the consensus diagnosis reached by the Geneva experts was 76.1% (kappa 0.28), which was poor.

### Diagnostic reliability

Table 4 represents the sensitivity and specificity of VIA and VILI combined. Sensitivity of the three Geneva physicians ranged from 50.0% (95%CI: 18.8–81.2) to 66.7% (95%CI: 30.0–90.3) and specificity ranged from 78.5% (95%CI: 68.2–86.1) to 83.8% (95%CI: 74.2–90.3).

**Table 4:** Sensitivity and specificity of VIA combined with VILI.

	Specificity % (95%CI)	Sensitivity % (95%CI)	PPV % (95%CI)	NPV % (95%CI)
**Onsite**	85.7 (76.7–91.6)	66.7 (30–90.3)	25 (10.2–49.5)	97.3 (90.7–99.3)
**Offsite Obs1**	83.8 (74.2–90.3)	66.7 (30–90.3)	23.5 (9.6–47.3)	97.1 (90–99.2)
**Offsite Obs2**	78.5 (68.2–86.1)	66.7 (30–90.3)	19.1 (7.7–40)	96.9 (89.3–99.1)
**Offsite Obs3**	82.1 (72.1–89)	50.0 (18.8–81.2)	17.7 (6.2–41)	95.5 (87.6–98.5)
**Offsite consensus**	82.3 (72.4–89.1)	66.7 (30–90.3)	22.2 (9–45.2)	97 (89.8–99.2)
**Telemedicine**	73.8 (63.5–82)	88.3 (43.7–97)	18.5 (8.2–36.7)	98.4 (91.5–99.7)

Abbreviations: CI = Confidence interval; PPV = Positive Predictive Value; NPV = Negative Predictive Value.

Consensus diagnosis (after case discussion between the three off-site physicians) reached a sensitivity of 66.7% (95%CI: 30.0–90.3) and a specificity of 82.3% (95%CI: 72.4–89.1). The sensitivity of the physician who performed analysis on-site was 66.7% (95%CI: 30.0–90.3) and the specificity was 85.7% (95%CI: 76.7–91.6). Telemedicine diagnosis consisted of the conjugation of on-site and off-site performances, and compared with the on-site performance, reached a sensitivity of 88.3% (95%CI: 43.7–97.0; p = 0.327) and a specificity of 73.8% (95%CI: 63.5–82.0; p = 0.005). Overall, three out of 95 (3.2%) photographs were considered inadequate for diagnosis.

## Discussion and Conclusions

The observed sociodemographic profile of the population included in the current study is typical of African populations and other areas in the world underserved by healthcare services, particularly in the observed low awareness of cervical cancer among the participants and the absence of screening [8–11]. This study has also shown a family history of cervical cancer to be a feature of some participants. Educational interventions, which increase knowledge and awareness of cervical cancer screening, may help increase screening uptake and the acceptability of HPV self-testing among women, thus working to improve women's health [12, 13].

There is enormous variability in the VIA method and recent data suggest this approach has intrinsic limitations, and training and quality control are mandatory for its success [3]. Using VIA is particularly challenging when it is used as primary screening test because the health care provider has to identify rare CIN2+ lesions (occurring in 2–4% of patients) among numerous benign changes [14].

To improve the efficiency and objectivity of the VIA approach, a primary self-HPV test was performed and only HPV-positive women were referred for VIA triage. For this group, the expected rate of CIN2+ increases, generally to between 8 and 12% [4, 15].

Quality assurance for VIA is a crucial issue and D-VIA could be a valuable adjunctive procedure [12]. Previous D-VIA screening used commercial digital cameras and the images were stored on laptop computers. Smartphones, with the ability to take high-quality images and to send information quickly, have a clear advantage over “standard” digital cameras: They are very easy to use, do not need an external light source and permit easy zooming in the photography as well as very quick and easy comparison of the different pictures taken (native, VIA and VILI). Experience has shown that smartphones have the capacity to monitor subtle cervical changes and to introduce a simple quality assurance process that can probably be easily integrated into a cervical cancer screening program [16–18].

In this pilot project, primary self-HPV testing was followed by VIA triage and D-VIA as quality control. The on-site physician had a sensitivity of 66.7% (95%CI: 30.0–90.3), which was identical to the off-site physician consensus 66.7% (95%CI: 30–90.3). Specificity was also similar for the detection of CIN2+, 85.7% (95%CI: 76.7–91.6) and 82.3% (95%CI: 72.4–89.1) for on-site and off-site physicians, respectively. These data show the similarity between results from on-site and off-site healthcare professionals, and suggest this model may be a promising approach for experts to support colleagues in remote areas.

A poor agreement between the on-site physician and the consensus diagnosis reached among the Geneva physicians (kappa 0.28) was observed. These data confirm that VIA interpretation can greatly vary between different observers. Subjectivity has also been reported in a study of telecolposcopy [17] for women living in rural areas, where the agreement between the on-site experts versus distant experts for positive colposcopic responses was 52.0% (kappa 0.23). Conversely, in a Botswanian study [19], the diagnostic concordance between nurse VIA and nurse photographic evaluation of VIA was 81%. Moderate agreement (kappa = 0.60) was also reported in a German study, where the primary and secondary examiners agreed in 69% of the cases [18]. Contrary to these studies, naked-eye visualization of the cervix instead of colposcopy was used in the present study, and this difference may have influenced the results.

Limitations of the current study include the small sample size and the significant drop-out rate (30.7%) among HPV-positive women. This may have been reduced if a “screen and treat” approach had been implemented. The availability of rapid point-of-care HPV testing might overcome this problem. By undertaking histopathological testing on all HPV-positive women, the sensitivity and specificity of on-site D-VIA and remote expert D-VIA could be directly compared with the pathological findings. A further positive aspect of this study is that only three (3.2%) of the photographs taken were of insufficient quality for diagnosis, which supports the reliability of image acquisition using a smartphone.

Generally, VIA method is intended to be performed by non-physician primary health care workers. In this study, a trained physician conducted the examination. Sensitivities between Geneva experts and the on-site physician were similar. The fact that the on-site physician was skilled in VIA may have masked the real potentialities of telemedicine. However, the goal of this study was to verify whether distant evaluation of D-VIA would be as accurate as on-site performance. We do believe these findings could be applied to other contexts where VIA is performed by non-physicians, since the essential is to take good quality images of the cervix by the VIA provider.

For taking adequate photographs of the cervix with the Samsung Galaxy S5 smartphone, several aspects need to be considered, as described before in the manuscript. In consequence, procedure standardization should be developed. This would involve standardization of shot angles, the distance from the cervix and the number of pictures taken. As previous studies have demonstrated, poor image quality or slight image manipulation may have an impact on the diagnostic accuracy [19, 20].

In the present study, the second reading of D-VIA was performed by physicians located in Switzerland. Remote healthcare services and technology are quickly becoming routine within healthcare institutions worldwide. In our case, once the program has been well implemented and is shown to be effective, it could be extended to other areas of Madagascar and even to other African countries. In the future, the possibility of the second D-VIA reading to be done by some expert located in an urban area of Madagascar or other African countries should be contemplated in order to combine screening with telemedicine. In our institution, a Telemedicine network (RAFT) [21] is already in place, where connections between university and district hospitals are established and participants come from different regions across the globe.

In conclusion, our data support the feasibility of telemedicine for detecting cervical intraepithelial neoplasia and cancer, with diagnostic accuracy similar to on-site results. Further studies are needed to determine if smartphones have the potential to improve cervical cancer screening efficiency in LMIC.
